# Cardiovascular magnetic resonance findings in a case of Danon disease

**DOI:** 10.1186/1532-429X-11-12

**Published:** 2009-04-29

**Authors:** Dorota Piotrowska-Kownacka, Lukasz Kownacki, Marek Kuch, Ewa Walczak, Agnieszka Kosieradzka, Anna Fidzianska, Leszek Krolicki

**Affiliations:** 1 II Department of Radiology, Medical University of Warsaw, Warsaw, Poland; 2 I Chair and Department of Cardiology, Medical University of Warsaw, Warsaw, Poland; 3 II Chair and Department of Cardiology, Medical University of Warsaw, Warsaw, Poland; 4 Department of Pathomorphology, Rheumatology Institute, Warsaw, Poland; 5 Neuromuscular Unit, Medical Centre, Polish Academy of Science, Warsaw, Poland; 6 Department of Nuclear Medicine, Medical University of Warsaw, Warsaw, Poland

## Abstract

Danon disease is a rare X-linked dominant lysosomal glycogen storage disease that can lead to severe ventricular hypertrophy and heart failure. We report a case of Danon disease with cardiac involvement evaluated with cardiovascular magnetic resonance, including late gadolinium enhancement and perfusion studies.

## Case presentation

A 19 year old male with no previous history of heart disease was admitted with rest dyspnoea, found to be due to acute heart failure. He had felt fatigued with progressive limitation of exercise tolerance over the preceding 3 months. Symptoms were exacerbated by an upper respiratory tract infection one month before hospitalization. The patient was treated with antibiotics without noticeable improvement. The patient's mother had died suddenly at the age of 44 with a dilated cardiomyopathy of unknown cause for which she had had a pacemaker implanted.

On admission, the patient was cachectic with a body mass index of 17 and in poor general condition, with rest dyspnoea, tachypnoea of 30/minute and tachycardia of 130/minute. His liver was enlarged, there was evidence of pulmonary oedema and a systolic murmur, maximal at the apex.

Blood analysis showed elevated liver enzymes (aspartate aminotransferase 192 units/L; alanine aminotransferase 400 u/L and creatine kinase 510 u/L) and mildly elevated Troponin I and C-reactive protein levels. Chest X-ray confirmed pulmonary oedema and showed an enlarged heart shadow. Sinus tachycardia and left bundle branch block with QRS duration >200 ms were present on electrocardiogram.

Echocardiography on admission revealed significantly enlarged left ventricle and both atria, severe hypertrophy of both ventricles muscle without left ventricular outflow tract (LVOT) obstruction. Moderate tricuspid and severe mitral valve insufficiency, decreased left ventricular (LV) ejection fraction to 30% with global hypokinesis were observed.

The patient was referred for cardiovascular magnetic resonance (CMR), which was performed on a 1.5 T system with 4 element torso coil. Oxygen was supplied by mask throughout the study at a flow rate of 3 l/min. Function was assessed with steady state free precession (SSFP) sequence in short axis slices covering the ventricles and in 4-chamber, 2-chamber and LVOT orientations. Perfusion was assessed at rest only in 8 short axis slices using a gradient echo sequence with inversion recovery during and after intravenous administration of Gadopentate dimeglumine (Gd-DTPA, 0,15 mmol/kg). Late gadolinium enhancement (LGE) images were obtained after 10–20 min in short axis and 4 chamber orientations. Inversion time was adjusted to null the signal from normal LV myocardium.

**Figure 1:**
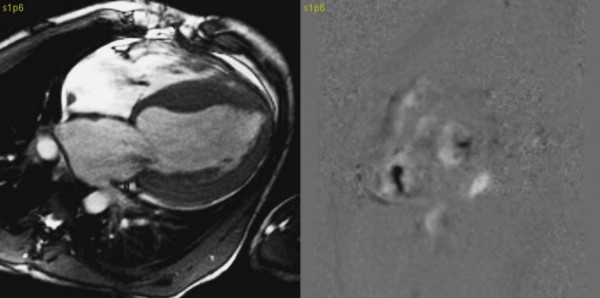
**On the left site SSFP image in 4 chamber orientation – signal lost due to tricuspid and mitral valves insufficiency**. On the right site velocity encoded phase contrast image in short axis orientation at the level of mitral and tricuspid valves – black spots indicate valves insufficiency.

The CMR study showed significantly reduced global function with left ventricular EF of 14%. (Additional file 1) Left ventricular end diastolic volume (EDV) and end systolic volumes (ESV) were increased (EDV 497 ml, ESV 426 ml). LV dimension was 81 × 94 mm in the short axis orientation. Significant dilatation of the right ventricle and both atria were confirmed. Tricuspid and mitral valve insufficiency were clearly visible on 4 chamber cine images and velocity encoded images in short axis orientation at valve level. (Figure 1)

**Figure 2:**
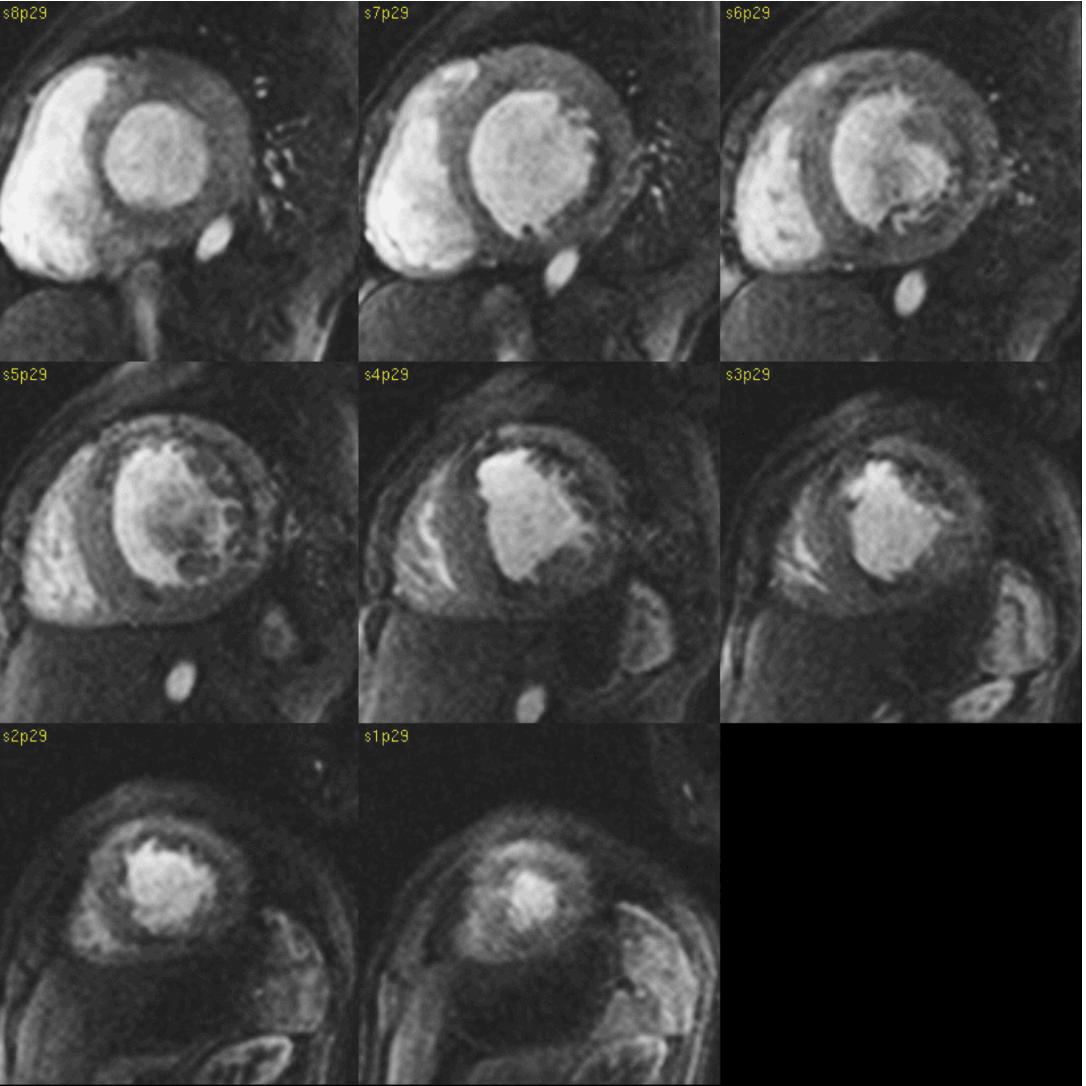
**First pass rest perfusion images in short axis orientation**. Subendocardial and partly transmural perfusion deficits visible clearly within anterior and later walls, RV inferior junction point.

**Figure 3:**
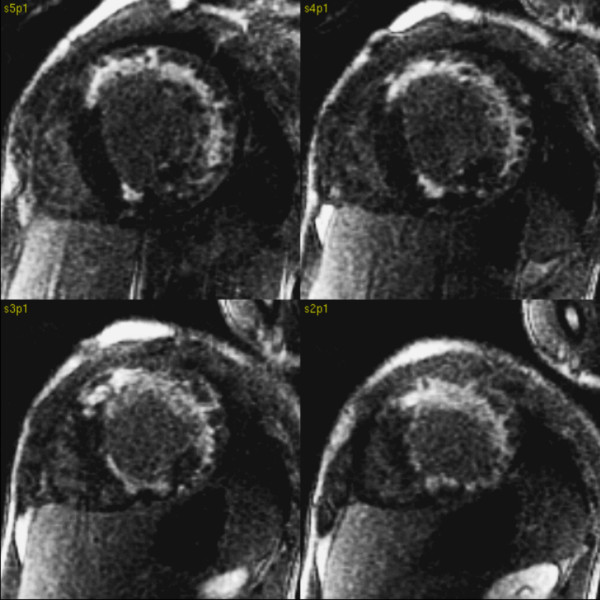
**Late gadolinium enhancement images in short axis orientation**. LGE was present in the subendocardium and the anterior and lateral wall, apical segments and RV inferior junction point.

Perfusion defects, mainly subendocardial, were visible in almost all segments on first pass images acquired at rest. They were obvious in the infero-septal segments and partly transmural in the lateral and anterior walls (Figure 2, Additional file 2). LGE was present in the subendocardium and in places transmurally in the anterior and lateral walls. A small LGE region was present in RV inferior junctional region. (Fig. 3)

The patterns of LGE and perfusion deficit were atypical for sarcomeric hypertrophic cardiomyopathy, and based on the CMR findings, myocarditis was considered unlikely. An ischaemic cause of the subendocardial LGE was considered, but the lack of correspondence with typical coronary territories and the combination with the severe hypertrophy of affected segments made this unlikely. Other causes of hypertrophy, including amyloidosis and Anderson-Fabry disease were also considered, but the LGE pattern did not seem typical. In Anderson-Fabry disease, the basal segments are predominantly affected. In amyloidosis, LGE can include subepicardial layers, but with a characteristic "zebra" pattern, and early decline of contrast levels in the blood stream.

**Figure 4:**
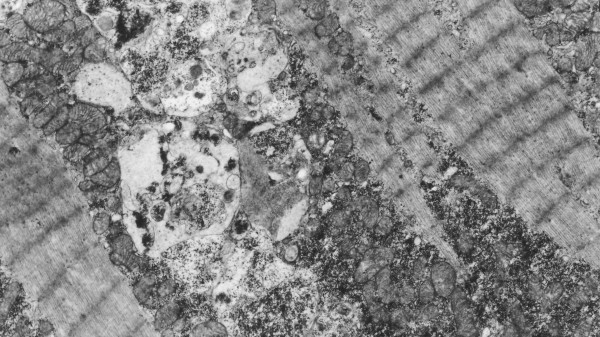
**Electron microscopic analysis of cardiomyocytes**. Autophagic vacuoles located within intramyofibrillar space × 10000

**Figure 5:**
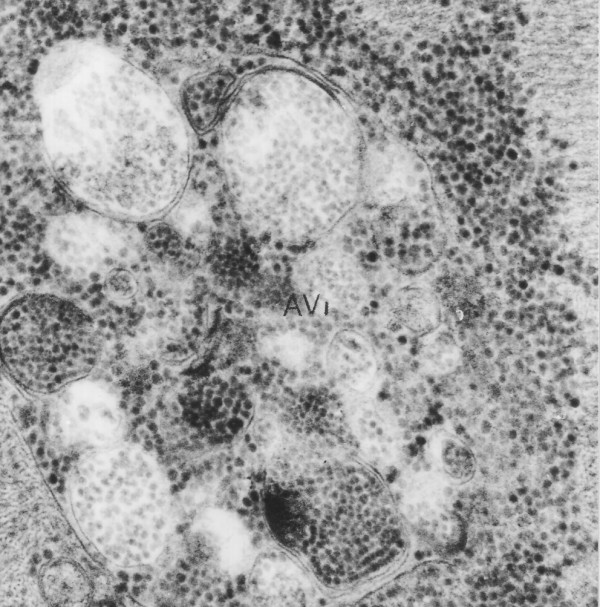
**Electron microscopic analysis of cardiomyocytes**. AVi vacuole filled with numerous glycogen enveloping structures. × 10000

**Figure 6:**
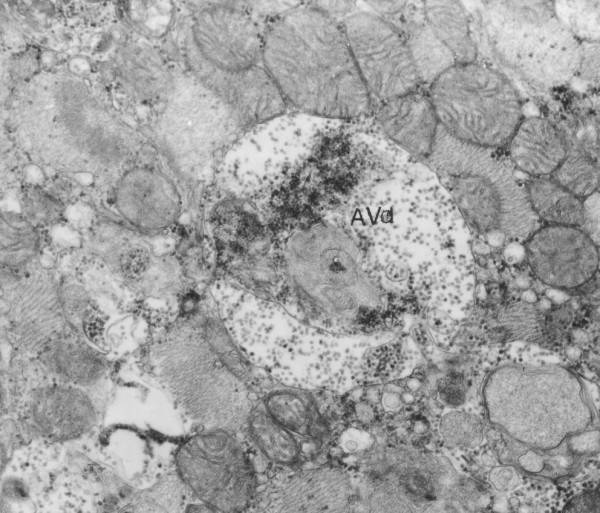
**Electron microscopic analysis of cardiomyocytes**. AVd limited by single membrane containing degraded material. × 20000

A diagnosis of Danon disease was confirmed by biopsy results. Skeletal muscle and endomyocardial biopsy from the right ventricle (RV) and the septum were taken. Electron microscopic analysis showed accumulation of autophagic vacuoles in affected cardiomyocytes. They were located within intrafibrillar spaces as well as in the perinuclear region (Figure 4). Many of these structures resembled early autophagic vacuoles (AVi) containing morphologically intact sarcoplasmatic contents (Figure 5) and double limiting membrane. A significant increase in the number of late autophagic vacuoles (AVd) limited by a single membrane and containing partially degraded sarcoplasm (Figure 6) suggests that their maturation is partially retarded. LAMP-2 protein deficiency which was detected by immunofluorescence study in striated muscle of the patient supported the diagnosis of Danon disease. The patient was treated for heart failure and placed on the heart transplantation list, but he died two weeks later.

Danon disease is a rare X – linked dominant, lysosomal glycogen storage disease that can lead to severe cardiac hypertrophy and heart failure especially in affected males. It was described first in 1981 in two boys by Moris J. Danon [1]. Mutation on LAMP2 gene, located on chromosome X, than encodes LAMP2 protein was identified as a cause of Danon Disease [2]. The X-linked disease should be considered in young males with cardiac hypertrophy and coexisting mental retardation/learning difficulties, skeletal myopathy or muscle weakness [3,4]. In some cases ophthalmic abnormalities [5] or WPW syndrome were reported [3,6]. Females who carry the mutation in the LAMP2 gene on chromosome X could develop dilated or hypertrophic cardiomyopathy in their early 40s. Mental retardation or skeletal myopathy can be present in female carriers, but less commonly than in affected males [4].

**Figure 7:**
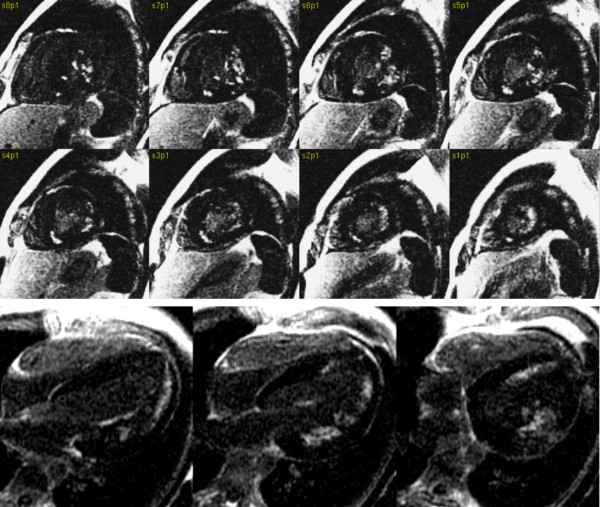
**Previously reported 24 yrs. old male with history of WPW syndrome treated with radio frequency (RF) ablation 3 years ago, with progressive muscle weakness, mental retardation **[7]. Late gadolinium enhancement images in short axis and 4 chamber orientation showed a subendocardial pattern of enhancement in the lateral wall, papillary muscle and RV inferior junction point. Perfusion deficits and late enhancement patterns were atypical for sarcomeric hypertrophic cardiomyopathy. The LGE pattern were similar to the patient in this case report. Based on CMR findings Danon disease was suspected. LAMP 2 protein deficiency was confirmed on immunofluorescence study of skeletal muscle which supported the diagnosis of Danon disease.

We present case of Danon disease confirmed by biopsy. As far as we know, this is the second description of CMR rest perfusion deficits and LGE in hypertrophic cardiomyopathy due to Danon disease [7]). In the case reported here, the LGE was mainly visible in the subendocardium and more extensively in the lateral segments in a distribution that would be unusual in more common pathologies [8]. Broadly similar LGE distribution and perfusion defects were seen in the previously described patient with preserved EF and only mild contractile dysfunction [7] (Figure 7). CMR examination with assessment of perfusion deficits and LGE can, in association with other investigations, be helpful in differential diagnosis of hypertrophic cardiomyopathy of unknown cause.

## Consent

Written informed consent was obtained from the deceased patient's next of the kin (sister) for publication of this case report and accompanying images. A copy of the written consent is available for review by the Editor-in-Chief of this journal.

## Competing interests

The authors declare that they have no competing interests.

## Authors' contributions

DPK and LKo contributed equally to this paper and should be considered joint first authors. They drafted the manuscript, performed CMR and interpreted CMR images. MK was patients cardiologists and wrote the clinical part of the manuscript. AK performed and interpreted ECHO. EW performed biopsy and established final diagnosis of Danon disease. EW and AF interpreted biopsy results. LKr helped write and rewrote the manuscript.

## Supplementary Material

Additional file 1 **Function**. SSFP cine images – short axis slices covering the whole ventricle Click here for file

Additional file 2 **Rest perfusion**. "First pass" rest perfusion in 8 SA slices. Subendocardial and partly transmural perfusion deficits visible clearly within anterior and later walls and RV inferior junction point. Click here for file
